# Reducing Generalization of Conditioned Fear: Beneficial Impact of Fear Relevance and Feedback in Discrimination Training

**DOI:** 10.3389/fpsyg.2021.665711

**Published:** 2021-06-01

**Authors:** Katharina Herzog, Marta Andreatta, Kristina Schneider, Miriam A. Schiele, Katharina Domschke, Marcel Romanos, Jürgen Deckert, Paul Pauli

**Affiliations:** ^1^Department of Psychology (Biological Psychology, Clinical Psychology, and Psychotherapy), Center of Mental Health, University of Würzburg, Würzburg, Germany; ^2^Department of Psychology, Educational Sciences, and Child Studies, Erasmus University of Rotterdam, Rotterdam, Netherlands; ^3^Department of Psychiatry and Psychotherapy, Medical Center—University of Freiburg, Faculty of Medicine, University of Freiburg, Freiburg, Germany; ^4^Department of Child and Adolescent Psychiatry, Psychosomatics, and Psychotherapy, Center of Mental Health, Würzburg, Germany; ^5^Department of Psychiatry, Psychosomatics, and Psychotherapy, Center of Mental Health, Würzburg, Germany

**Keywords:** fear generalization, feedback, discrimination training, fear-relevant training, classical conditioning

## Abstract

Anxiety patients over-generalize fear, possibly because of an incapacity to discriminate threat and safety signals. Discrimination trainings are promising approaches for reducing such fear over-generalization. Here we investigated the efficacy of a fear-relevant vs. a fear-irrelevant discrimination training on fear generalization and whether the effects are increased with feedback during training. Eighty participants underwent two fear acquisition blocks, during which one face (conditioned stimulus, CS+), but not another face (CS−), was associated with a female scream (unconditioned stimulus, US). During two generalization blocks, both CSs plus four morphs (generalization stimuli, GS1–GS4) were presented. Between these generalization blocks, half of the participants underwent a fear-relevant discrimination training (discrimination between CS+ and the other faces) with or without feedback and the other half a fear-irrelevant discrimination training (discrimination between the width of lines) with or without feedback. US expectancy, arousal, valence ratings, and skin conductance responses (SCR) indicated successful fear acquisition. Importantly, fear-relevant vs. fear-irrelevant discrimination trainings and feedback vs. no feedback reduced generalization as reflected in US expectancy ratings independently from one another. No effects of training condition were found for arousal and valence ratings or SCR. In summary, this is a first indication that fear-relevant discrimination training and feedback can improve the discrimination between threat and safety signals in healthy individuals, at least for learning-related evaluations, but not evaluations of valence or (physiological) arousal.

## Introduction

The adaptive mechanism of fear generalization prevents the encounter with unknown threats by extending previous learning to new cues (Dunsmoor and Paz, 2015). Thus, fear responses are elicited not only by cues (conditioned stimulus, CS+) predicting threat (i.e., the unconditioned stimulus, US; LeDoux and Pine, 2016) but also by stimuli, which were never associated with the US but share physical or semantical properties with the threat-associated cues (Lissek et al., 2008; Dunsmoor and Paz, 2015; Dymond et al., 2015). Notably, this mechanism is exaggerated in anxiety patients compared to healthy controls, leading to over-generalization of fear (Lissek et al., 2010, 2014). Similarly, high trait-anxiety individuals, i.e., individuals at risk for anxiety disorders (Raymond et al., 2017), tend to over-generalize conditioned fear (Baumann et al., 2017; Stegmann et al., 2019; but see Torrents-Rodas et al., 2013; for a meta-analysis, see Sep et al., 2019).

Generalization of conditioned fear seems to be related to an incapacity in perceptually discriminating the relevant stimuli (Holt et al., 2014; Struyf et al., 2018; Zaman et al., 2019). For example, Holt et al. (2014) first assessed the participants’ ability to discriminate the to-be-conditioned face from other faces, i.e., they assessed the just noticeable differences (JND) between CS+ vs. CS− and their morphs (i.e., generalization stimuli, GSs). After differential conditioning, a generalization test revealed increased US expectancy for the GSs below and at JND, indicating generalization, but not for those above JND. Thus, generalization of conditioned fear did not occur to those GSs, which were reliably discriminated from the CS+. Accordingly, improving the discrimination of CS+ may be a promising approach to reduce fear generalization.

Following this idea, several kinds of discrimination training have been developed to prevent fear generalization (Ginat-Frolich et al., 2017, 2019; Lommen et al., 2017). Specifically, two studies applied a discrimination training of fear-irrelevant stimuli (Ginat-Frolich et al., 2017, 2019). Each discrimination training trial started with a 4-s presentation of one abstract shape (target) at the center of a screen. Then, the target plus a new similar shape were presented on the left and right of the screen, and the participants had to identify the target. This training group was compared to a non-discriminative control task group where the participants had to indicate on which side of the screen each shape appeared. As a result, the authors observed reduced fear generalization of US expectancy in the first compared to the latter group.

Another study applied a discrimination training with stimuli sharing one perceptual characteristic (i.e., color) with the CS+ (Lommen et al., 2017). The participants had to decide if two stimuli presented shortly after each other differed in size or color or not at all. The participants assigned to a control task had to indicate instead which of two words was related to each presented stimulus. In contrast, this discrimination training which occurred before fear acquisition did not reduce the fear generalization of US expectancy but of avoidance behavior, again compared to the non-discriminative control task. These studies suggest that discrimination trainings with either fear-irrelevant or partial fear-relevant stimuli reduce fear generalization. However, please note that these trainings occurred before (Lommen et al., 2017) or after (Ginat-Frolich et al., 2017, 2019) fear learning. Therefore, only the latter two studies reflect a treatment training, while the former mirrors a prevention training.

One limitation of the above-mentioned studies is that they have tested the effects of discrimination training before having examined the generalization gradient of their participants. However, anxiety disorder patients start treatments after fear acquisition and with a long history of over-generalization. Thus, we examined the fear-reducing effects of a discrimination training after both fear acquisition and a first demonstration of fear generalization. Our study was designed in order to reduce fear generalization, which occurred due to problems with perceptually discriminating CS+ from safe similar stimuli. As there is evidence that perceptual learning cannot be easily transferred to an unpracticed stimulus set (Furmanski and Engel, 2000), we examined whether discrimination trainings with fear-relevant vs. fear-irrelevant stimuli show higher efficacy. Additionally, we examined whether feedback on discrimination performance further enhances training efficiency. The latter expectation is based on previous studies demonstrating that positive feedback (Sasaki et al., 2009) on participants’ performance and, in particular, when associated with reward (Weil et al., 2010) is able to improve stimulus perception.

To examine these hypotheses, our participants first underwent differential fear conditioning followed by two generalization blocks separated by discrimination training. Importantly, participants either learned to discriminate fear-relevant (discrimination of CS+ from GSs and CS−) or fear-irrelevant (discrimination of a specific line width from other lines) stimuli either with or without performance feedback. We hypothesized that the fear-relevant training reduces fear generalization more effectively than the fear-irrelevant training, and that this effect is especially strong if feedback is applied.

## Materials and Methods

### Participants

The participants were recruited *via* an internet platform of the University of Würzburg. The exclusion criteria were psychiatric and neurological disorders, intake of psychoactive medication, excessive consumption of alcohol or nicotine, and pregnancy. Moreover, only participants between the ages of 18 and 50 were included. They were screened by a telephone interview. In the end, 80 participants were randomly divided into four groups (for details, see Table 1). Before the experiment started, all the participants read and signed the informed consent. The study was approved by the Ethical Committee of the Medical Board of the University of Würzburg and was conducted in accordance with the ethical principles of the Helsinki Declaration.

**TABLE 1:** Descriptive statistics of the four groups.

	Relevant_DT _noFB	Relevant_DT _FB	Irrelevant_DT _noFB	irrelevant_DT _FB	Comparisons
*N*	20	20	20	20	
Gender (♀)	10	10	10	10	
Age (SD)	24.25 (4.27)	26.10 (8.42)	25.70 (7.40)	24.75 (5.37)	*F*_(1,76)_ = 0.91, *p* = 0.344
Language	17 German	17 German	18 German	19 German	χ(3) = 2.16, *p* = 0.540
Handedness	19 right-hand	19 right-hand	19 right-hand	17 right-hand	χ(1) = 0.72, *p* = 0.396
STAI (SD)	34.30 (9.14)	32.70 (6.53)	34.90 (10.18)	35.25 (7.41)	*F*_(1,76)_ = 0.27, *p* = 0.607
BDI (SD)	5.1 (5.91)	3.70 (4.46)	5.25 (6.21)	5.55 (6.35)	*F*_(1,76)_ = 0.43, *p* = 0.513

### Stimulus Material

Two neutral face expressions of a brunette and a blond woman (03F_NE_C, 10F_NE_C, NimStim Face Stimulus Set, Tottenham et al., 2009) served as conditioned stimuli (CS). One face stimulus (CS+) was associated with the aversive unconditioned stimulus (US) in 83% of the trials, while the other face (CS−) was never associated with the US (see also “Procedure”). The faces were counter-balanced across participants.

A compound of a 95-dB female desperate scream (International Affective Digitized Sounds, IADS, FemScream2, No. 276; Bradley and Lang, 1999) and the woman’s fearful face expression were presented at the offset of CS+ for 1.5 s as US.

Four gradual morphs of the CSs were created in 20% steps by means of the software Squirlz Morph (for details, see Schiele et al., 2016, version 2.1, Xiberpix, Solihull, United Kingdom) and used as generalization stimuli (GS). The CSs and GSs were presented for 6 s each.

After each block (see “Procedure”), the participants indicated the arousal (“how much stress/tension/arousal was elicited by this stimulus?”) and valence (“how pleasant vs. unpleasant was the stimulus for you?”) of each face on Likert scales from 1 (“calm” or “very unpleasant”) to 9 (“intense” or “pleasant”). Then, US expectancy ratings (“how high is the probability that you will hear the scream by this stimulus?”) were asked using a Likert scale ranging from 0 (“very unlikely”) to 100 (“very likely”), except after the habituation phase. The faces were rated in a fixed order (brunette woman, blond woman, morphs ranging from brunette to blond woman) and were presented for 1 s each before a Likert scale appeared.

For sample description and assessment of emotional state, the participants were asked to fill in selected questionnaires (for details, see Supplementary Material).

### Procedure

In the laboratory, the participants were seated on a comfortable chair, and they filled in the questionnaires. All stimuli were presented using Presentation software, version 16.0 (Neurobehavioral Systems, Inc., Albany, CA). The participants were instructed to passively view pictures and that they will occasionally hear an unpleasant loud sound, but the CS–US contingency was not disclosed.

**FIGURE 1:**
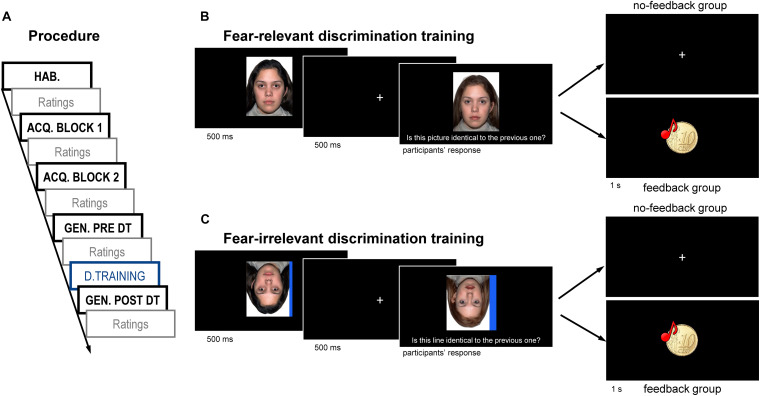
Overview of the experimental procedure **(A)** as well as of the fear-relevant discrimination **(B)** and fear-irrelevant discrimination **(C)** trainings, both with and without feedback. The discrimination training was surrounded by two generalization blocks, pre- and post-discrimination training, and the participants were divided into four groups: two groups underwent a fear-relevant discrimination training, and the other two groups underwent a fear-irrelevant discrimination training. Firstly, the participants saw a stimulus (CS+ for the fear-relevant training, a line for the fear-irrelevant training) for 500 ms, which was always the same. After 500 ms of inter-stimulus interval, a second stimulus was presented until the participants indicated whether the second stimulus was identical with the first stimulus or not. The second stimulus was for the fear-relevant discrimination training, the CS+, the CS–, or any of the generalization stimuli, and for the fear-irrelevant discrimination training a line with the same or a different width. Half of the participants received reinforcing feedback for correct answers (feedback groups; feedback for correct answers was a compound stimulus of a 10-Euro-cent picture and a cash register sound lasting 1 s and no feedback for incorrect answers), while the other half never received feedback (no-feedback groups). The face pictures presented here have been obtained from the NimStim Face Stimulus Set by Tottenham et al. (2009), some of which were further processed by morphing. They are examples and differ from the pictures used.

The experimental procedure (Figure 1A) was based on previous studies (Lau et al., 2008; Schiele et al., 2016). Stimuli were presented in a pseudo-randomized order so that the same stimulus appeared not more than twice in a row. During inter-trial-intervals (ITI, time between two stimulus presentations), a white fixation cross was displayed in the center of the screen for 9–12 s randomly. During the habituation phase, both the CS+ and the CS− were presented four times each without any US.

The acquisition phase and generalization phase were divided into two identical blocks containing six presentations of each stimulus. In the acquisition blocks, US was delivered at CS+ offset in five trials out of six and never after CS−. During the generalization blocks, i.e., generalization tests, all faces were presented, and in three CS+ trials the US was delivered to prevent the extinction of conditioned fear. Between the generalization blocks, the participants underwent discrimination trainings (see below).

After each block, the participants rated the arousal and valence of the faces and indicated their US expectancy (except after the habituation phase). Throughout the whole experiment, skin conductance responses (SCRs) were recorded. After the experiment, the participants completed the state questionnaires again.

### Discrimination Trainings

The training protocol was inspired by the discrimination task described previously (Holt et al., 2014). In all trainings, the faces and morphs were equally presented.

For the fear-relevant discrimination training (Figure 1B), every trial began with a 500-ms CS+ presentation. After a 500-ms inter-stimulus interval, a second stimulus was presented, which was either CS+, one GS, or CS−, below which the participants could read the question “Is this picture identical with the previous one?” The participants had to respond by pressing the S (yes) or L (no) button on the German keyboard with no time limitation. The face and question lasted on the screen until a response was given.

One group of participants (relevant_DT_FB) received reinforcing feedback for correct answers, consisting of a 10-Euro cent picture and a cash register sound (52.4 dB) lasting 1 s. No feedback was provided for incorrect answers. Another group (relevant_DT_noFB) received no feedback.

During the fear-irrelevant discrimination training (Figure 1C), all faces were turned upside down, and a blue line (35–79 pixels in width) was presented on the right or left side. Importantly, the width of the first stimulus line was always the thinnest line and was always combined with the upside-down CS+ face. All other aspects were as described for the fear-relevant discrimination training, and the participants had to indicate whether the width of the lines was identical or not. One group (irrelevant_DT_FB) received feedback again, but not the other group (irrelevant_DT_noFB).

In all trainings, the ITI lasted 1–3 s randomly. The participants altogether performed 50 training trials with a short break after 25 trials. For 20 out of the 50 trials, the second stimulus was the CS+ or the thinnest line, i.e., the pictures were identical. For the remaining 30 trials, the two pictures were not identical; each of the remaining five faces or lines were presented five times. While the thin line of the first stimulus was always combined with the upside down CS+, the line–face combinations of the second stimulus varied. Nevertheless, as during the fear-relevant training, in the fear-irrelevant training every non-CS+ face was presented five times each.

Unfortunately, we cannot report the training performance of the two no-feedback groups due to a programming error. However, the training performances of the two feedback groups are reported in Supplementary Material.

### Data Recording and Reduction

Throughout the experiment, SCRs were recorded with two 8-mm Ag/AgCl electrodes attached at the thenar and hypothenar eminences of the non-dominant hand. For recording, Brainproducts V-Amp and BrainVision Recorder software (version 1.21, Brainproducts, Gilching, Germany) were used, having a sampling rate of 1,000 Hz and an online notch filter of 50 Hz. Offline analyses were run with BrainVision Analyzer Software (version 2.1, Brainproducts, Gilching, Germany). The electro-dermal signal was first filtered with a high cutoff filter of 1 Hz. In accordance with the guidelines (Boucsein et al., 2012), SCRs were defined as the difference in μS between response onset (900–4,000 ms after stimulus onset) and peak (2,000–6,000 ms after stimulus onset). Reactions smaller than 0.02 μS were set to 0. Next, every reaction of each participant was range-corrected, i.e., divided by the individual’s strongest reaction to a face picture, i.e., CS or GS. Besides this, all SCRs were log-transformed into log_10_(SCR + 1). The mean values were then calculated for each stimulus and experimental block. The participants (*n* = 5) with an overall raw mean response smaller than 0.02 μS were considered as non-responders and therefore excluded from the statistical testing of SCR. Accordingly, *n* = 20 of the relevant_DT_FB group, *n* = 16 of the relevant_DT_noFB group, *n* = 19 of the irrelevant_DT_FB group, and *n* = 20 of the irrelevant_DT_noFB group were included in the statistical analysis of SCR.

### Statistical Analysis

Statistical analyses were carried out in the R software environment (version 3.6.1) using the packages “afex” (version 0.26-0; Singmann et al., 2015) and “emmeans” (version 1.4.5; Lenth and Lenth, 2018).

Fear acquisition effects were analyzed with ANOVAs having stimulus (CS+, CS−) and block as within-subject factors; the factor block had three levels (habituation, acquisition 1, and acquisition 2) for arousal, valence, and SCR data and two levels (acquisition 1 and acquisition 2) for US expectancy data.

The discrimination training effects were investigated as follows: First, we calculated a generalization index (GI) for each generalization block defined as the sum of all GS responses divided by the CS+ response: GI = [(GS1 + GS2 + GS3 + GS4)/CS+] (for details, see Lenaert et al., 2016). In other words, the GI represents the reaction to all GSs relative to CS+. As our fear-relevant discrimination training focuses on the discrimination of all GSs from CS+, the GI fits perfectly to reveal training-related changes in discrimination. To prevent division by zero, all US expectancy values were increased by 10 (i.e., the smallest step on the US expectancy scale), and all values of SCR were increased by log(0.02 + 1) (defined as smallest SCR reaction >0, see above). Then, ANCOVAs on GI post-training were calculated, with fear relevance (relevant_DT, irrelevant_DT) and feedback (with, without) as between-subjects factors and GI pre-training as covariate. The ANCOVA is a commonly used method for comparing pre–post change across groups because of its good statistical power and only a slight bias for floor effects (Jennings and Cribbie, 2016).

The alpha level was set at.05, and Greenhouse–Geisser correction was applied for violation of the sphericity assumption. Where necessary, Bonferroni-corrected simple contrasts were calculated as *post hoc* tests. For effect sizes, partial eta-square values are reported.

## Results

### Acquisition of Conditioned Fear

**FIGURE 2:**
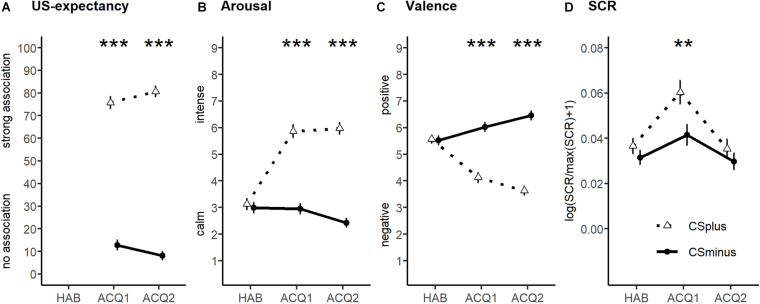
Habituation and acquisition ratings of unconditioned stimulus (US) expectancy **(A)**, arousal **(B)**, and valence **(C)** as well as skin conductance responses **(D)**. Means (with SEs) of ratings and skin conductance responses (SCRs) are depicted for CS+ (white) and CS– (black) for habituation (HAB) for acquisition blocks (ACQ1, ACQ2). Acquisition of conditioned fear was successful as the CS+ vs. the CS– was rated as more likely predicting the US, more arousing, and more negatively valenced, and this elicited larger SCRs. Significance symbols indicate *post hoc* simple contrasts; ***p < 0.001, **p < 0.01.

Successful fear acquisition is indicated by significant stimulus × block interactions for all dependent variables, i.e., US expectancy [*F*_(1,79)_ = 19.41, *p* < 0.001, $\eta_{p}^{2}$ = 0.20; Figure 2A], arousal [*F*_(1.89,149.12)_ = 86.34, *p* < 0.001,$\eta_{p}^{2}$ = 0.52; Figure 2B], and valence ratings [*F*_(1.86,.146.81)_ = 82.83, *p* < 0.001,$\eta_{p}^{2}$ = 0.51; Figure 2C] and for SCR [*F*_(1.31,96.87)_ = 6.25, *p* = −0.008, $\eta_{p}^{2}$ = 0.08; Figure 2D]. *Post hoc* contrasts confirmed that the ratings were higher for CS+ vs. CS− regarding US expectancy [acquisition1: *F*_(1,79)_ = 343.24, *p* < 0.001, $\eta_{p}^{2}$ = 0.81; acquisition2: *F*_(1,79)_ = 673.82, *p* < 0.001,$\eta_{p}^{2}$ = 0.90; Bonferroni-corrected α < 0.025], arousal [acquisition1: *F*_(1,79)_ = 98.12, *p* < 0.001,$\eta_{p}^{2}$ = 0.55; acquisition2: *F*_(1,79)_ = 165.31, *p* < 0.001,$\eta_{p}^{2}$ = 0.68; Bonferroni corrected α < 0.017], and valence [acquisition1: *F*_(1,79)_ = 59.85, *p* < 0.001,$\eta_{p}^{2}$ = 0.431; acquisition2: *F*_(1,79)_ = 129.52, *p* < 0.001,$\eta_{p}^{2}$ = 0.621; Bonferroni corrected α < 0.017], while no CS+ vs. CS− differences were evident for the habituation phase [arousal: *F*_(1,79)_ = 0.62, *p* = 0.434,$\eta_{p}^{2}$ = 0.01; valence: *F*_(1,79)_ = 0.05, *p* = 0.829,$\eta_{p}^{2}$ < 0.01]. *Post hoc* contrasts for SCR also revealed no CS+ vs. CS− differences for habituation phase [*F*_(1,74)_ = 2.24, *p* = 0.139,$\eta_{p}^{2}$ < 0.03]. Regarding the acquisition phase, physiological arousal was higher to CS+ vs. CS− for acquisition 1 [*F*_(1,74)_ = 11.81, *p* < 0.001, $\eta_{p}^{2}$ = 0.14], but not for acquisition 2 [*F*_(1,74)_ = 1.50, *p* = 0.225, $\eta_{p}^{2}$ = 0.02], possibly due to habituation effects.

These ANOVAs also returned significant main effects of stimulus for all dependent variables [US expectancy: *F*_(1,79)_ = 538.75, *p* < 0.001,$\eta_{p}^{2}$ = 0.87; arousal: *F*_(1,79)_ = 124.05, *p* < 0.001,$\eta_{p}^{2}$ = 0.61; valence: *F*_(1,79)_ = 73.29, *p* < 0.001,$\eta_{p}^{2}$ = 0.48; SCR: *F*_(1,72)_ = 7.12, *p* = 0.009,$\eta_{p}^{2}$ = 0.09] and main effects of block for arousal [*F*_(1.54,121.81)_ = 45.51, *p* < 0.001,$\eta_{p}^{2}$ = 0.377], valence [*F*_(1.63,129.00)_ = 14.08, *p* < 0.001, $\eta_{p}^{2}$ = 0.15], and SCR [F_(1,72)_ = 24.44, *p* < 0.001,$\eta_{p}^{2}$ = 0.06], but not for US expectancy [*F*_(1,79)_ = 0.01, *p* = 0.907, $\eta_{p}^{2}$ < 0.01].

### Discrimination Training Effects

**FIGURE 3:**
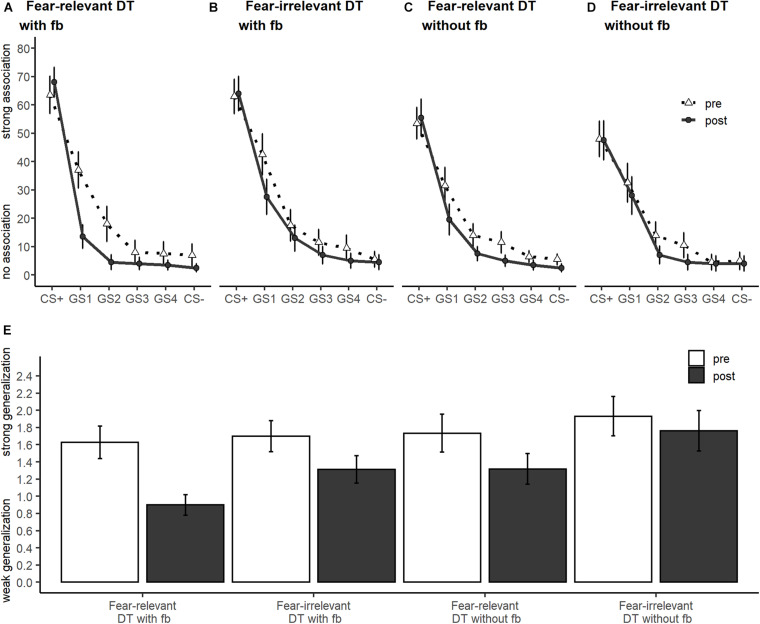
Generalization gradients **(A–D)** and generalization indices **(E)** for unconditioned stimulus expectancy ratings separately for each training group and generalization pre- and post-training.

**FIGURE 4:**
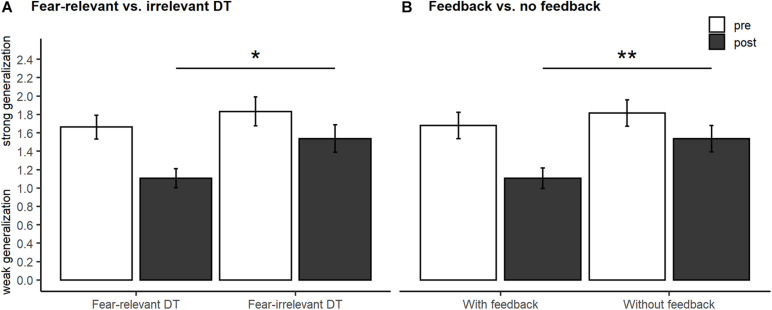
Generalization indices (GI) for unconditioned stimulus expectancy ratings for generalization pre- and post-training averaged by factor fear relevance **(A)** or feedback **(B)**. Bar plots (with means and standard errors) of GI show less fear generalization post-training in the fear-relevant vs. fear-irrelevant discrimination training groups **(A)** and in the groups with vs. without feedback (fb, **B**). The significance symbols indicate the main effects of ANCOVA. *p < 0.05, **p < 0.01.

For US expectancy ratings (the corresponding generalization gradients are depicted in Figures 3A–D), the ANCOVA on GIs revealed significant main effects of training [*F*_(1, 75)_ = 6.56, *p* = 0.012, $\eta_{p}^{2}$ = 0.08; Figure 4A] and feedback [*F*_(1, 75)_ = 7.27, *p* = 0.009, $\eta_{p}^{2}$ = 0.09; Figure 4B], but not their interaction [*F*_(1, 75)_ = 0.04, *p* = 0.850, $\eta_{p}^{2}$ < 0.01; Figure 3E], indicating that fear generalization was reduced more effectively by fear-relevant or rewarding feedback conditions.

**FIGURE 5:**
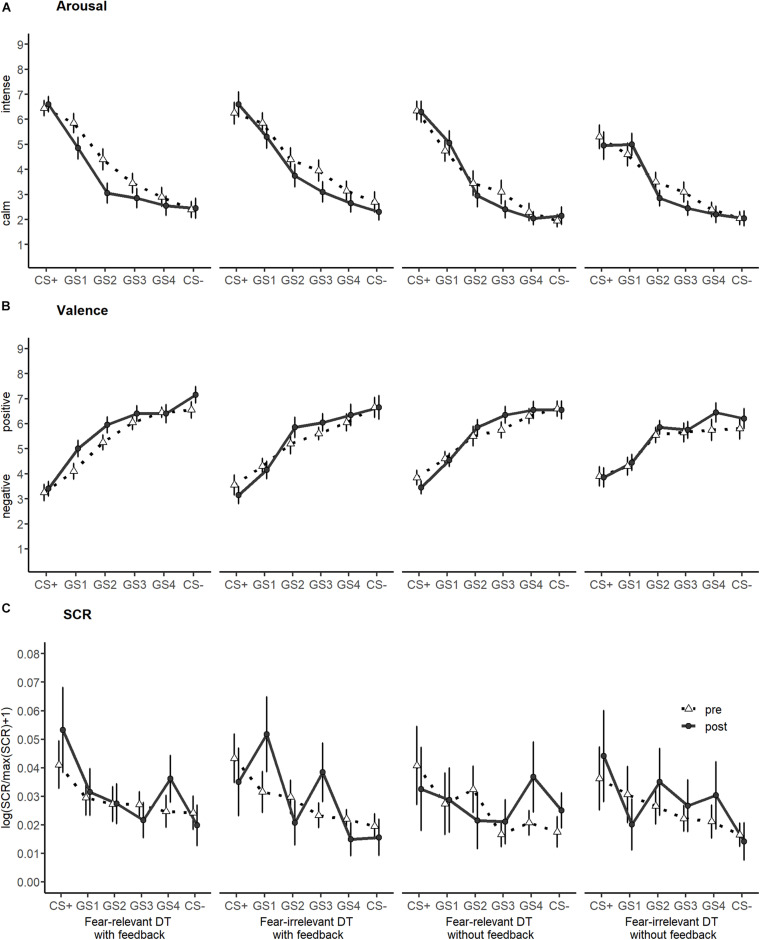
Generalization gradients for arousal **(A)** and valence ratings **(B)** as well as skin conductance responses **(C)** separately for each training group and generalization pre- and post-training.

For arousal and valence ratings (for means and SDs, see Table 2; the corresponding generalization gradients are depicted in Figures 5A,B), the ANCOVAs on GIs revealed no effects involving the between-factors (all *p* > 0.106).

**TABLE 2:** Generalization indices for arousal and valence ratings as well as skin conductance responses, separately for each training group and generalization pre- and post-training.

	Relevant_DT	Irrelevant_DT	Relevant_DT	Irrelevant_DT
	_FB	_FB	_noFB	_noFB
**Arousal**				
Pre (SD)	2.60 (0.70)	2.95 (1.09)	2.15 (0.83)	2.69 (0.76)
Post (SD)	2.11 (1.04)	2.51 (1.45)	2.05 (0.82)	3.26 (2.55)
**Valence**				
Pre (SD)	2.81 (0.87)	3.41 (2.64)	2.98 (0.74)	3.23 (0.94)
Post (SD)	2.58 (1.01)	2.71 (1.04)	2.62 (0.79)	3.11 (1.11)
**SCR**				
Pre (SD)	3.78 (2.48)	4.15 (3.05)	4.23 (3.01)	5.34 (4.28)
Post (SD)	5.80 (5.71)	9.83 (10.52)	7.63 (6.90)	5.41 (5.23)

The main effect of covariate was significant for all ratings [US expectancy: *F*_(1, 75)_ = 72.13, *p* < 0.001, $\eta_{p}^{2}$ = 0.49; arousal: *F*_(1, 75)_ = 9.71, *p* = 0.003, $\eta_{p}^{2}$ = 0.11; valence: *F*_(1, 75)_ = 14.92, *p* < 0.001, $\eta_{p}^{2}$ = 0.17], suggesting positive associations between generalizations assessed pre- and post-training [US expectancy: *r*(79) = 0.69, *p* < 0.001; arousal: *r*(79) = 0.35, *p* = 0.001; valence: *r*(79) = 0.72, *p* < 0.001].

The analysis of SCR with ANCOVA on GIs (see Table 2; the corresponding generalization gradients are depicted in Figure 5C) revealed a marginal significant interaction of fear relevance and feedback [*F*_(1, 70)_ = 3.7, *p* = 0.059, $\eta_{p}^{2}$ = 0.05] only (all other *p* > 0.153). Bonferroni-corrected (α < 0.012) *post hoc* simple contrasts did not confirm a significant impact of the combination of fear relevance and feedback (all *p* > 0.043).

## Discussion

This study was designed to test our hypothesis that the generalization of conditioned fear to perceptually similar stimuli can be reduced significantly better by a discrimination training with fear-relevant vs. fear-irrelevant stimuli. Additionally, we hypothesized that discrimination training effects profit by reinforcing feedback with greatest effects due to fear-relevant training with feedback. The experimental design followed the logic of a treatment, which normally starts in patients with ascertained fear generalization. Thus, the discrimination training occurred after fear acquisition and in between two generalization tests. In consequence, this is the first study allowing one to verify training effects by comparing the generalization indices pre- vs. post-training.

Analysis of acquisition data validated that successful fear conditioning as the threat cue (CS+) vs. the safety cue (CS−) triggered greater arousal, more negative valence, greater US expectancy, and larger SCRs. Furthermore, analyses of the CS+ vs. CS− responses during the generalization tests pre- and post-discrimination training (see Supplementary Material) indicate that these conditioned fear responses remained stable throughout the experiment. Overall, these results corroborate previous studies using similar paradigms (Lissek et al., 2008; Schiele et al., 2016) and indicate the successful acquisition of fear toward the CS+.

Analyses of discrimination training effects on fear generalization suggest, on the one hand, stronger effects for the fear-relevant vs. the fear-irrelevant discrimination training and, on the other hand, stronger effects for discrimination training with reinforcing feedback vs. without feedback, but with no interaction of effects. Thus, we conclude that a reduction of fear generalization is especially effective when the discrimination training is carried out with fear-relevant stimuli vs. fear-irrelevant stimuli or with reinforcing feedback vs. no feedback. However, we found no support for our hypothesis that a combination of training, i.e., training with fear-relevant stimuli *and* feedback, is especially effective in reducing fear generalization. Please note, however, that these findings are restricted to US expectancy ratings.

These results raise the following questions: Firstly, what contributes to the observed superior effect of a discrimination training with fear-relevant vs. fear-irrelevant stimuli on reducing fear generalization? Secondly, why is feedback during discrimination training effective in reducing fear generalization independently from the training stimuli? Thirdly, why are the observed discrimination training effects restricted to the US expectancy ratings?

Regarding the first question, the effect might be related to working memory (WM) processes, which are addressed during discrimination trainings as the participants have to keep in mind the first stimulus in order to compare it with the subsequent one (Lenaert et al., 2016). Considering that WM filters irrelevant from relevant information (Eysenck et al., 2007; Derakshan and Eysenck, 2009; Baddeley, 2012), it is very likely that a fear-relevant discrimination training improves the discrimination between relevant and irrelevant CS+ information, especially strong, and subsequently reduces the generalization effects. Through its relevance in attentional control (Eysenck et al., 2007; Derakshan and Eysenck, 2009), WM might facilitate perceptual learning (Amitay et al., 2014), possibly also thanks to inductive processes like the derivation of rules from WM (e.g., paying attention to visual features of stimuli; Livesey and McLaren, 2009). Rule learning processes might be further promoted by the fixed sequence of stimulus presentations during training (always beginning with CS+ or Line1), which could lead to an overestimation of perceptual learning (García-Pérez and Alcalá-Quintana, 2020). Secondly, the results go in line with previous reports that a discriminative training with fear-relevant stimuli is able to reduce fear generalization (Lommen et al., 2017), although this study trained the participants before fear acquisition in analogy to prevention, while we trained the participants after fear acquisition in analogy to therapy. Moreover, it goes in line with more general findings regarding the transfer of discrimination learning to a test phase, which was found to be more effective after training relevant compared to irrelevant stimuli (Furmanski and Engel, 2000). In our study, the fear-relevant and fear-irrelevant training groups both have improved discrimination performance during training (for more details, see Supplementary Material). Consequently, the more efficient fear reduction of the fear-relevant training group might be related to a more effective transfer to the post-training generalization test. However, we know from previous studies that discrimination training, also with fear-irrelevant stimuli, reduces subsequent generalization effects in general (Ginat-Frolich et al., 2017, 2019). This can explain why the effects of fear-relevance are present but rather small.

Regarding the second question, we argue that feedback affects very general processes unrelated to the processing and discrimination of the presented stimuli. Previous studies revealed that feedback improves performance in general (Sasaki et al., 2009; Weil et al., 2010) as well as mood (Westermann et al., 1996). Moreover, reward facilitates attentional processes toward the reward-associated cue (Chelazzi et al., 2013) and increases motivation (Fishbach et al., 2010). Therefore, we assume that the implemented reinforcing feedback improved our participants’ mood, attention, and motivation in general, which consequently improved their discrimination during the following generalization test. To verify this hypothesis, future studies might consider adding a control condition without discrimination training but a motivation condition, e.g., a task with comparable cognitive load and reinforcing feedback for correct responses.

Thirdly, we have to consider why the effects of the implemented discrimination trainings were restricted to US expectancy ratings. One possible explanation lies in the typology of the ratings. As previously suggested (Lonsdorf et al., 2017), US expectancy reflects cognitive learning processes, while valence and arousal ratings rather reflect affective learning processes. Therefore, new learning experiences can be seen more easily and earlier in US expectancy ratings. In line with this, the beneficial effects of our training were visible for US expectancy ratings. In contrast, extinction after evaluative conditioning, reflected in changes in valence, is hard to achieve (Vansteenwegen et al., 2006; Hofmann et al., 2010). This might also be true for discrimination learning, which could explain why we did not find training effects for the affective ratings. Moreover, the dissociation between affective and cognitive ratings suggests that, apart from perception, other (higher-order) processes (e.g., WM and inference rules) might have also determined fear generalization and its reduction in our paradigm. We suggest that future studies should explore whether and at what level an extensive discrimination training can improve discrimination as reflected in affective responses and, in consequence, reduce generalization.

In order to reveal discrimination training effects on the fear generalization of affective responses, future studies might consider assessing online ratings of valence and arousal during tests. We assessed the ratings at the end of every generalization block only and therefore could not determine discrimination training effects by comparing ratings from the end of generalization pre-training with the beginning of generalization post-training. Considering that online ratings might draw attention to US contingencies (Lonsdorf et al., 2017), we decided to assess intermittent ratings as several previous studies did (Haddad et al., 2012; Meulders et al., 2013; Holt et al., 2014; Roesmann et al., 2020).

Finally, we want to address the limitations of the current study. First, one limitation concerns the potential role of extinction and safety learning as additional mechanisms contributing to the higher efficacy of fear-relevant discrimination training. During the fear-relevant training, the subjects are exposed to the CS+ and the GSs without the US’s presence, likely triggering extinction and safety learning processes. We can assume that our measured effects do not include extinction processes for CS+ because USs were presented again during generalization testing, thereby very likely undoing the CS+ extinction learning (as indicated by stable conditioned fear response to CS+ both pre- and post-training; see Supplementary Material). However, we cannot rule out that lower responses to certain GSs (and in consequence less generalization as reflected in the generalization index) stem from safety learning rather than some effects of perceptual discrimination training. Therefore, future studies should consider examining an additional group that did not undergo discrimination training but was exposed to the same amount of stimuli to control for potential extinction and safety learning effects. Second, no effects of fear relevance or feedback could be confirmed for physiological arousal, i.e., SCRs, which limits the effectiveness of our “therapeutic” approach of discrimination training to the verbal level. However, previous studies also did not find training effects for physiological measures (Ginat-Frolich et al., 2017) or did not include physiological measures (Lommen et al., 2017). In our study, contrary to all ratings, SCRs post-training were not significantly influenced by the pre-training level, as shown by the absent main effect of covariate. This means that all trainings strongly influenced the electrodermal activity in a way that dissolved the pre-training generalization pattern. Conceivably, the main reason lies in the nature of a training. While the participants passively observed the stimuli during the generalization blocks, during the discrimination training they actively fulfilled a task. The active task might have overlaid specific training effects. Besides this, the discriminative physiological arousal found during the first acquisition block disappeared in the second acquisition block, which is presumably due to habituation and could explain the absence of training effects for SCR. Third, the sample sizes are rather small. It should be considered, however, that the study presented here is a proof-of-principle study to establish a therapeutical approach of fear reduction by discrimination trainings. Fourth, we examined healthy participants who show moderate fear generalization as previous studies have also reported (Lissek et al., 2008). Considering the over-generalization in highly anxious individuals and anxiety patients (Lissek et al., 2010, 2014; Laufer et al., 2016; Sep et al., 2019), future studies should investigate discrimination training in more clinic-relevant samples. Fifth, we have to acknowledge that, due to a programming error, we have no data on the discrimination training performance for the no-feedback groups. Therefore, we are unable to test whether and how feedback improved discrimination performance during training, and consequently, we cannot relate feedback effects on performance to later generalization effects.

In conclusion, this proof-of-principle study demonstrated the successful reduction of existing fear generalization by a fear-relevant discrimination training in healthy individuals at least for cognitive fear parameters, i.e., US expectancy. Moreover, we revealed that reinforcing feedback during discrimination training reduces the generalization of US expectancy, presumably *via* motivational mechanisms. Importantly, our study is the first to mimic a therapeutic approach as the discrimination training was performed after fear acquisition and a first demonstration of fear generalization. Accordingly, this is the first indication that pre-existing fear generalization can be reduced by means of a fear-relevant discrimination training or a reinforcing discrimination feedback, which in the future might be successfully used in patients with anxiety disorders.

## Data Availability Statement

The raw data supporting the conclusions of this article will be made available by the authors, without undue reservation.

## Ethics Statement

The studies involving human participants were reviewed and approved by the Ethical Committee of the Medical Board of the University of Würzburg, Würzburg, Germany. The patients/participants provided their written informed consent to participate in this study.

## Author Contributions

KH, MA, KD, MR, JD, and PP contributed to conception and design of the study. KS was involved in the conception and design of the discrimination training. MS contributed to the design of the fear generalization paradigm. KH organized the database and performed the statistical analysis. KH, MA, and PP wrote the first draft of the manuscript and critically revised each section. All authors contributed to manuscript revision, read, and approved the submitted version.

## Conflict of Interest

The authors declare that the research was conducted in the absence of any commercial or financial relationships that could be construed as a potential conflict of interest.
