# Supplementary material for: Reducing Generalization of Conditioned Fear: Beneficial Impact of Fear Relevance and Feedback in Discrimination Training
Source: Front Psychol. 2021 Jun 1;12:665711. doi: 10.3389/fpsyg.2021.665711 (PMC8203828; doi:10.3389/fpsyg.2021.665711)
Supplement: Supplementary file 1 [file Table_1.DOCX]

Supplementary Material

# Supplementary Data

## Sample description and questionnaires

The group descriptions are summarized in Table 1. We found no pre-experimental differences between groups.

For a broader sample description, participants filled in the German version of the State-Trait Anxiety Inventory (STAI; Laux, Glanzmann, Schaffner, & Spielberger, 1981) as well as of the Beck-Depression Inventory (Hautzinger, Keller, & Kühner, 2006) before the experiment (Table 1). As state control measures, the state version of the STAI and the Positive and Negative Affective Schedule (PANAS, Krohne, Egloff, Kohmann, & Tausch, 1996) were completed at the beginning and the end of the experimental protocol (Supplementary Table 1 and Supplementary Table 2).

The 2 (phase: beginning, end of experiment) x 2 (training: relevant_DT, irrelevant_DT) x 2 (feedback: with, without) ANOVAs indicate a significant main effect for phases for all three scales (Supplementary Table 1; state anxiety: *F*(1,76) = 27.28, *p* < .001, $\eta_{p}^{2}$ = .264; negative mood: *F*(1,76) = 20.96, *p* < .001, $\eta_{p}^{2}$ = .216; positive mood: *F*(1,76) = 11.46, *p* = .001, $\eta_{p}^{2}$ = .131) meaning that participants were more anxious (*F*(1,76) = 27.28, *p* < .001, $\eta_{p}^{2}$ = .264) and had higher negative mood (*F*(1,76) = 20.96, *p* < .001, $\eta_{p}^{2}$ = .216) as well as lower positive mood (*F*(1,76) = 11.46, *p* = .001, $\eta_{p}^{2}$ = .131) at the end of the experiment as compared to the beginning. Moreover, we observed a significant interaction between training and feedback for the negative mood scale (*F*(1,76) = 5.72, *p* = .019, $\eta_{p}^{2}$ = .070), and significant Phase x Training x Feedback interaction (Supplementary Table 2) for state anxiety (*F*(1,76) = 4.63, *p* = .035, $\eta_{p}^{2}$ = .057) and negative mood (*F*(1,76) = 7.47, *p* = .008, $\eta_{p}^{2}$ = .089), but not positive mood (*F*(1,76) = 0.15, *p* = .704, $\eta_{p}^{2}$ = .002). No further effects were found (all *p* values > .069). Post-hoc simple contrasts (Bonferroni corrected, α < .012) for the three-way interaction indicated that the participants receiving fear-irrelevant discrimination training with feedback had higher state anxiety (*F*(1,19) = 16.83, *p* = .001, $\eta_{p}^{2}$ = .470) and higher negative mood (*F*(1,19) = 13.95, *p* = .001, $\eta_{p}^{2}$ =.423) at the end of the experiment as compared to the beginning, while all other groups showed no significant change of their state anxiety or mood throughout the experiment (all *p* values > .016)

## Discrimination performance during training

The discrimination training performance, i.e. percentage of mistakes, was analyzed with an ANOVA having the within-subject factors learning (Part1, Part2) and comparison (i.e. CS+ - CS+, CS+ -GS1, CS+ -GS2, CS+ -GS3, CS+ -GS4, CS+ -CS- in the relevant_DT group, or rather Line1-Line1, Line1-Line2, Line1-Line3, Line1-Line4, Line1-Line5, Line1-Line6 in the irrelevant_DT group) as well as the between-subjects factor fear-relevance (relevant_DT, irrelevant_DT), but not feedback because of missing data.

The ANOVA on discrimination performance during training showed a marginal Learning x Comparison interaction (*F*(1.97, 74.72) = 2.74, *p* = .072, $\eta_{p}^{2}$ = .07, Supplementary Table 3), indicating that the capacity to discriminate either the CS+ or the thin line from the other stimuli changed differently during the two discrimination training parts. Post-hoc simple contrasts revealed a lower percentage of mistakes in Part 2 compared to Part 1 for one comparison only, namely the comparison of CS+ or the Line1 with itself (*F*(1, 38) = 6.88, *p* = .006, $\eta_{p}^{2}$ = .15, all other *p*-values > .077, Bonferroni corrected $\alpha$ < .008). Besides, we found a main effect of comparison (*F*(2.44, 92.62) = 39.79, *p* < .001, $\eta_{p}^{2}$ = .51, Supplementary Table 3). Post-hoc simple contrasts (Bonferroni corrected $\alpha$ < .010) indicated that during the complete training participants made more incorrect discriminations between the CS+ or the Line1 and the CS+ or the Line1 respectively (*F*(1, 38) = 15.32, *p* < .001, $\eta_{p}^{2}$ = .29) as well as between CS+/Line1 and GS1 or Line2 respectively (*F*(1, 38) = 65.83, *p* < .001, $\eta_{p}^{2}$ = .63) as compared to between CS+/Line1 and CS- or Line6 respectively. The percentage of mistakes of all other comparisons did not differ from that with CS- or Line6 (all *p*-values > .067). Moreover, the fear-relevant discrimination training had a lower percentage of mistakes compared to the fear-irrelevant alternative, as indicated by the main effect of fear-relevance (*F*(1, 38) = 14.12, *p* < .001, $\eta_{p}^{2}$ = .27, *M*_fear-relevant_ = 5.80, *SD*_fear-relevant_ = 4.30, *M*_fear-irrelevant_ = 15.30, *SD*_fear-irrelevant_ = 10.96). No other effect reached significance (all *p*-values > .229).

Against our expectations, the fear-relevant training group demonstrated a better performance as indicated by lower error rates than the fear-irrelevant group. Possibly, participants of the fear-relevant group had the advantage to have seen the stimuli previously (i.e., during the first generalization block) and such familiarity might have increased the discrimination performance. Despite both fear-relevant and fear-irrelevant groups showed an improvement in their discrimination performance throughout the training, the error rate for the comparison of CS+ with GS1 or Line1 with Line2 remained high throughout the training. This result was unexpected as the discrimination between these stimuli should have improved throughout the training as well. Compared to the comparisons of CS+ or Line1 with itself, the number of learning trials for both the CS+/GS1 and the Line1/Line2 was quite reduced, i.e. participants were asked only six times to compare these stimuli. Consequently, we presume that these six trials were not sufficient to improve such complex or difficult discrimination as GS1 and Line2 shared 80% of the physical properties with CS+ and Line1 respectively. Nevertheless, the trainings improved discrimination in general, which was then transferred to the subsequent generalization test, especially by the fear-relevant training group. This brings up the question, whether the training of reliable recognition of threat and its features, instead of discrimination from threat, could be another approach to reduce exaggerated fear generalization.

## Stability of responses to conditioned stimuli throughout the experiment

We performed ANOVAs having the within-subject factors stimulus (CS+, CS-) and training (pre, post) to examine specifically the “stability” of fear learning during the generalization trainings.

ANOVAs on CS responses during generalization tests pre and post training returned significant main effects of stimulus for all dependent variables (US-expectancy: *F*(1, 79) = 325.43, *p* < .001, $\eta_{p}^{2}$ = .80, CS+: *M* = 57.88, *SD* = 28.20, CS-: *M* = 4.56, *SD* = 11.65; arousal: *F*(1, 79) = 251.48, *p* < .001, $\eta_{p}^{2}$ = .76, CS+: *M* = 6.10, *SD* = 1.98, CS-: *M* = 2.26, *SD* = 1.48; valence: *F*(1, 79) = 116.12, *p* < .001, $\eta_{p}^{2}$ = .60, CS+: *M* = 3.55, *SD* = 1.52, CS-: *M* = 6.52, *SD* = 1.71; SCR: *F*(1, 72) = 18.83, *p* < .001, $\eta_{p}^{2}$ = .21; CS+: *M* = 0.04, *SD* = 0.05, CS-: *M* = 0.02, *SD* = 0.02), indicating stability of the conditioned responses throughout both generalization test. The Stimulus x Training interaction was significant for US-expectancy (*F*(1, 79) = 4.24, *p* = .043, $\eta_{p}^{2}$ = .05, CS+_pre_: *M* = 57.00, *SD* = 27.94, CS-_pre_: *M* = 5.75, *SD* = 13.39, CS+_post_: *M* = 58.75, *SD* = 28.61, CS-_post_: *M* = 3.38, *SD* = 9.54) and trendwise for valence ratings (*F*(1, 79) = 3.04, *p* = .085, $\eta_{p}^{2}$ = .04, CS+_pre_: *M* = 3.64, *SD* = 1.59, CS-_pre_: *M* = 6.40, *SD* = 1.66, CS+_post_: *M* = 3.46, *SD* = 1.46, CS-_post_: *M* = 6.64, *SD* = 1.77). No other effects were found (all *p* values > .642). Post-hoc contrasts (Bonferroni corrected α < .012) of these interactions indicate stable conditioned fear responses as CS+ versus CS- ratings were higher both at pre (US-expectancy: *F*(1, 79) = 278.17, *p* < .001, $\eta_{p}^{2}$ = .78, valence: *F*(1, 79) = 84.25, *p* < .001, $\eta_{p}^{2}$ = .52) and post training generalization tests (US-expectancy: *F*(1, 79) = 305.73, *p* < .001, $\eta_{p}^{2}$ = .79, valence: *F*(1, 79) = 113.05, *p* < .001, $\eta_{p}^{2}$ = .59). We also observed no significant changes in the ratings for CS+ or CS- from pre to post training assessments (all *p* values > .184), with the exception of a decrease in US-expectancy ratings to CS- from pre to post training assessment (*F*(1, 79) = 8.8, *p* = .002, $\eta_{p}^{2}$ = .10).

# Supplementary Tables

| **Supplementary Table 1.** Changes in the emotional state of the participants throughout the experimental procedure independently from the task. | | | | | | |
| --- | --- | --- | --- | --- | --- | --- |
|  | *STAI state* | | *Negative Mood* | | *Positive Mood* | |
| begin (*SD*) | 32.90 (6.37) | | 11.50 (2.25) | | 31.74 (5.85) | |
| end (*SD*) | 36.51 (8.06) | | 13.05 (3.31) | | 30.08 (6.36) | |
| **Supplementary Table 2.** Changes in the emotional state of the participants throughout the experimental procedure dependent on the task. | | | | | | |
|  | *relevant_DT*  *_noFB* | *relevant_DT*  *_FB* | | *irrelevant_DT*  *_noFB* | | *irrelevant_DT*  *_FB* |
|  | ***STAI state*** | | | | | |
| begin (*SD*) | 34.40 (6.11) | 33.80 (5.83) | | 31.10 (7.38) | | 32.30 (6.00) |
| end (*SD*) | 38.05 (6.64) | 35.65 (5.62) | | 33.50 (8.12) | | 38.85 (10.48) |
|  |  |  | |  | |  |
|  | **Negative Mood** | | | | | |
| begin (*SD*) | 11.95 (2.06) | 11.35 (2.30) | | 11.35 (1.98) | | 11.35 (2.70) |
| end (*SD*) | 13.50 (3.15) | 11.65 (1.63) | | 12.30 (2.39) | | 14.75 (4.61) |
|  |  |  | |  | |  |
|  | **Positive Mood** | | | | | |
| begin (*SD*) | 31.15 (4.60) | 33.80 (5.28) | | 31.45 (6.07) | | 30.55 (7.06) |
| end (*SD*) | 29.30 (5.21) | 32.10 (5.78) | | 29.45 (6.63) | | 29.45 (7.60) |
|  | | | | | | |

| **Supplementary Table 3.** Discrimination performance for each demand level of the training indicated by mistakes (percent). | | | | | | |
| --- | --- | --- | --- | --- | --- | --- |
|  |  |  |  |  |  |  |
|  | ***CS+ - CS+*** | ***CS+ - GS1*** | ***CS+ - GS2*** | ***CS+ - GS3*** | ***CS+ - GS4*** | ***CS+ - CS-*** |
|  | ***or*** | ***or*** | ***or*** | ***or*** | ***or*** | ***or*** |
|  | ***Line1 - Line1*** | ***Line1 - Line2*** | ***Line1 - Line3*** | ***Line1 - Line4*** | ***Line1 - Line5*** | ***Line1 - Line6*** |
| part 1 (SD) | 17.00 (24.93) | 28.33 (27.79) | 8.33 (21.01) | 5.83 (16.69) | 3.33 (16.54) | 5.00 (14.22) |
| part 2 (SD) | 6.00 (8.10) | 37.50 (35.56) | 5.00 (14.22) | 3.33 (12.63) | 0.83 (5.27) | 1.67 (7.36) |
|  |  |  |  |  |  |  |
| total (SD) | 11.50 (12.82) | 32.92 (23.72) | 6.67 (14.52) | 4.58 (10.67) | 2.08 (8.60) | 3.33 (8.61) |
